# Medicinal plant use, conservation, and the associated traditional knowledge in rural communities in Eastern Uganda

**DOI:** 10.1186/s41182-022-00428-1

**Published:** 2022-06-06

**Authors:** Jamilu E. Ssenku, Shaban A. Okurut, Aidah Namuli, Ali Kudamba, Patience Tugume, Paul Matovu, Godfrey Wasige, Hussein M. Kafeero, Abdul Walusansa

**Affiliations:** 1 Department of Biological Sciences, Faculty of Science, Islamic University in Uganda, Mbale, Uganda; 2 Department of Microbiology and Immunology, Faculty of Health Sciences, Habib Medical School, Islamic University in Uganda, Kampala, Uganda; 3 Department of Plant Sciences, Microbiology and Biotechnology, Makerere University, Kampala, Uganda; 4 Forestry Sector Support Department, Nyabyeya Forestry College, Ministry of Water and Environment, Masindi, Uganda

**Keywords:** Traditional medicine, Herbal medicine conservation, Indigenous medicinal knowledge, Uganda, Butaleja district

## Abstract

**Background:**

The global consumption of herbal medicine is increasing steadily, posing an extinction risk to medicinal plants. Uganda is among the top ten countries with a high threat of herbal medicine extinction, and Traditional Medicinal Knowledge (TMK) erosion. This might be attributed to the inadequate documentation, plus many more unclear hindrances. In this study, plant species used to treat human diseases in Butaleja district in Eastern Uganda and their associated TMK were documented. The conservation methods for medicinal plants were also evaluated. The rationale was to support the preservation of ethnopharmacological knowledge.

**Methods:**

Data were collected from 80 herbalists using semi-structured questionnaires, from July 2020 to March 2021. Additionally, guided field walks and observations were conducted. Quantitative indices such as, use categories and informant consensus factor (ICF) were evaluated to elucidate the importance of the medicinal plants. Data were analyzed using STATA version-15.0 software.

**Results:**

In total, 133 species, belonging to 34 families and 125 genera were identified. Fabaceae (65%), and Solanaceae (29%) were the dominant families. Leaves (80%), and roots (15%), were the commonest parts used in medicinal preparations; mostly administered orally as decoctions (34.6%) and infusions (16%). The commonest illnesses treated were cough (7.74%), gastric ulcers (7.42%), and malaria (4.52%). The informant consensus factor was high for all disease categories (≥ 0.8), indicating homogeneity of knowledge about remedies used. Only 73% of the respondents made efforts to conserve medicinal plants. The commonest conservation strategy was preservation of forests with spiritually valued species (100%), while compliance with government regulations was the rarest (4.5%). Overall, efforts to stop the extinction of medicinal plants and TMK were inadequate.

**Conclusion and recommendations:**

There was enormous dependency on a rich diversity of medicinal plant species and TMK for healthcare and income generation. The potential for medicinal plant biodiversity loss was evident due to habitat destruction. Inclusion of traditional cultural norms in conservation strategies, and laboratory-based efficacy tests for the species identified are necessary, to promote the conservative and utilization of validated herbal medicines and TMK in rural settings.

**Supplementary Information:**

The online version contains supplementary material available at 10.1186/s41182-022-00428-1.

## Background

Plants have been used for their therapeutic properties since antiquity [1, 2]. Consequently, human medicine is much linked to plant biodiversity and the fact that most of the current modern medicines were first derived from medicinal plants [3]. Across centuries, the global rate of medicinal plants use has been increasing, more so in developing countries [4]. This is mostly attributed to the fact that medicinal plants are readily available, widely accepted by many cultures, and perceived to be safe and efficacious [5]. At present, the estimated prevalence of global medicinal plants’ use ranges between 50 and 95% [6, 7]. Due to this high demand for herbal medicine in both developing and developed countries and other factors such as climate change, about 15,000 medicinal plant species worldwide are at risk of extinction [8, 9]. Currently, over 20% of the wild medicinal plant species are already nearly exhausted [10].

In Uganda, the prevalence of herbal medicine use was estimated to be 60% [4, 11]. The consumption is generally higher in rural areas, partly due to limited access to medical facilities; inadequacy and high cost of medical supplies, shortage and low motivation of medical practitioners, and wide acceptance of traditional medicines as having minimal side effects compared to conventional drugs [12, 13]. In Uganda, medicinal plants are mainly collected from the wild using destructive harvesting techniques such as uprooting and debarking that endangers their survival [14]. Inappropriate harvesting, and consumption of herbal medicine is partly fueled by the increasing proliferation of unregulated herbal medicine trade in urban centers. The indiscriminate collection and overharvesting of medicinal plant species for commercial purposes threatens the conservation of plant diversity [15].

In the recent past, Uganda has been listed among the countries that are facing the greatest risk of future extinction of medicinal plants [15, 16]. Unfortunately, this coincided with Uganda’s efforts towards upscaling herbal medicine use and its prospective integration into the main health care system [17, 18]. Butaleja, is a remote district in eastern Uganda with relatively poor infrastructure and service delivery. It is deficient in pharmacies, licensed private drug shops, and affordable private clinics [19]. Therefore, the district is characterized by widespread use of medicinal plants for disease treatment and health promotion [4, 20, 21]. Unfortunately, erosion of traditional medicinal knowledge (TMK) and threats to biodiversity conservation, such as; climate changes and widespread herbal medicine trade [20], have been reported in this area [20, 22]. We present results that reveal the medicinal plant species, their associated TMK, and approaches used in their conservation in Butaleja district. The findings highlighted the need for sustainable utilization and conservation of medicinal plant species and TMK in this area and similar rural communities in the tropics. This could protect future generations against dire health consequences of medicinal plants extinction.

## Materials and methods

### Study area

**Fig. 1:**
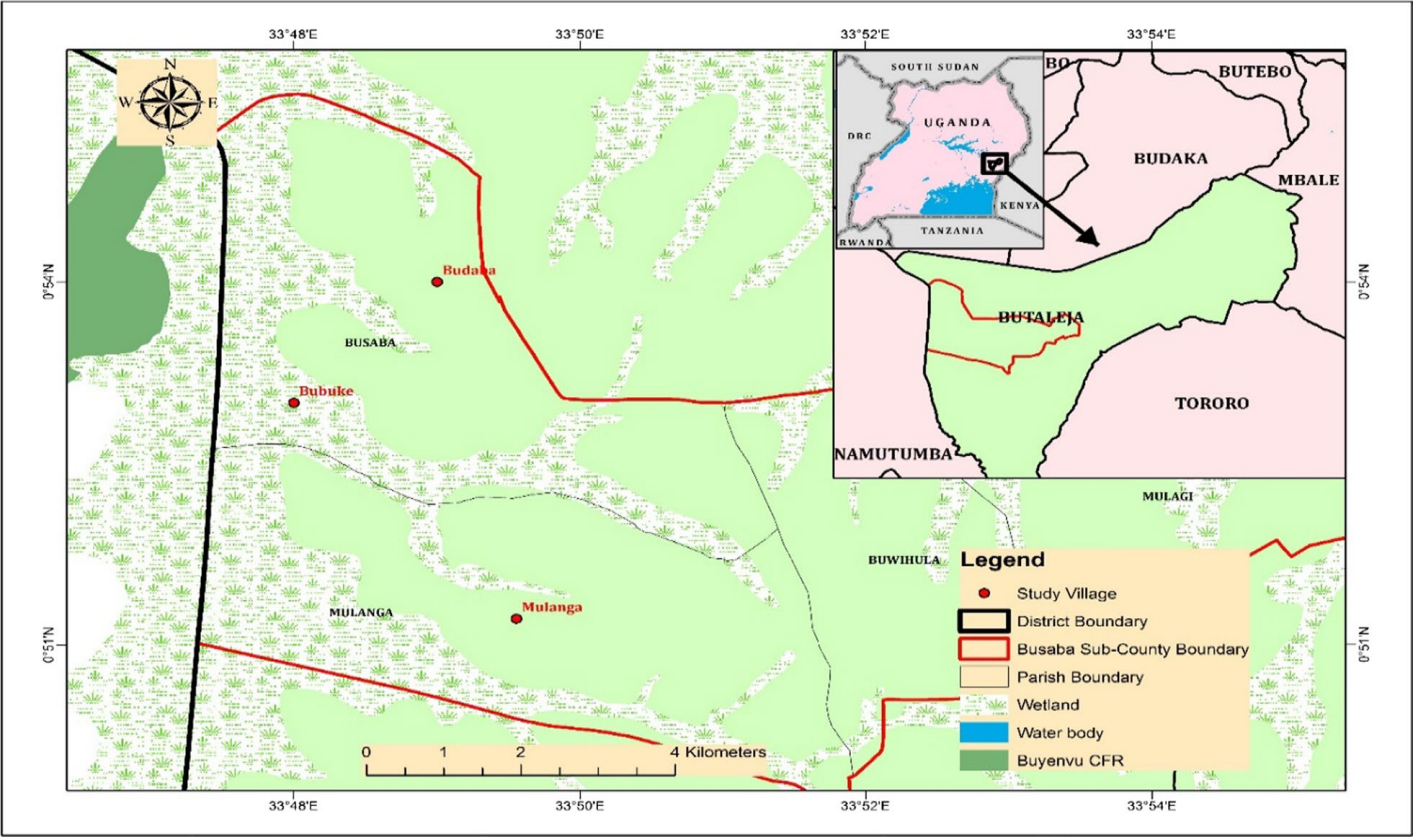
Location of the study area

The study was conducted in three villages namely; Budoba, Bubuhe and Mulanga, located in Busaba sub-county, in Butaleja district. Communities in the selected villages have limited access to medical facilities and thus heavily dependent on medicinal plants for primary health care. The district is located in the remote part of Eastern Uganda, 38 km from Mbale city and 247.8 km from Kampala, the country’s capital and major commercial city. Butaleja district lies between of 33° 45″ E to 33° 53″ E and 0° 51″ N to 0° 55″ N (Fig. 1) and at an average elevation of 1143 m above sea level. It experiences a tropical climate, and receives an annual average rainfall of 1183 mm, that is bimodally distributed with the highest peaks occurring in April–June and October–December. The mean monthly temperature ranges between 10 °C and 27 °C. The vegetation majorly comprises wetlands, few thickets, grasslands and forests. Considerable changes in vegetation are taking place due to widespread cultivation of food crops such as rice and population pressure.

### Demographic characteristics of the population

The majority of the population belongs to the Banyole ethnic group and the main language spoken is Lunyole. The community is both rural and semi urban, characterized by relatively high levels of poverty and illiteracy [23]. Most people are peasant farmers majorly involved in wetland-based rice growing and small-scale businesses for livelihoods.

### Study design and sampling technique

The study was conducted between July 2020 and March 2021 in conformity to the national guidelines for conducting research in the COVID-19 era established by the Uganda National Council for Science and Technology [24]. A cross-sectional study design was adopted to conduct an ethnopharmacological survey, using semi-structured questionnaires and Key informant interviews. Eighty renown Traditional health practitioners (THP), were purposively identified from the three villages, first under the guidance of local council chairpersons; and then through snowball sampling. The Participants were subjected to pretested, semi-structured questionnaires and key informant interviews in their local language (Lunyole). For each medicinal plant species, the respondents were asked to provide the local name, the ailment treated, part used, mode of administration, availability, conservation strategies, and associated TMK, plus the challenges. We calculated the percentage citation of a particular disease using the formula:

$$\mathrm{Percentage} \, \mathrm{citation}=\frac{\mathrm{Total}\, \mathrm{citations}\, \mathrm{of} \,\mathrm{a} \,\mathrm{disease}}{\mathrm{Total}\, \mathrm{citations}\, \mathrm{for} \,\mathrm{all}\, \mathrm{diseases}}\times 100.$$

The non-generic names were either in Lunyole (Lun) and Luganda (Lug). With the help of key informants, voucher specimens of all mentioned medicinal plant species were collected and prepared following standard procedures [25]. Species identification was done at Makerere University Herbarium and the correctness of the scientific names was verified using the Plant List database [26], accessed on 15th May 2021. The voucher specimens were deposited at the Islamic University in Uganda Herbarium. The filled questionnaires were checked for consistency and completeness before final data processing and analysis.

### The informant consensus factor

The ailments treated were categorized basing on the eight systems of the human body, cause of disease, and the medication characteristics of the people of Butaleja. The informant consensus factor (ICF) was then calculated to assess the homogeneity in the ethnomedicinal information documented from the traditional health practitioners based on diseases treated and plant species used [27] using the following formula [28].

$$\mathrm{IFC}=\frac{{N}_{\mathrm{ur}}-{N}_{t}}{{N}_{\mathrm{ur}}-1},$$

where *N*_ur_ is the total number of plant-use reports (citations), for a disease category, and *N*_t_ is the total number of plant species used for that ailment category.

### Data analysis

Simple descriptive statistics were adopted for data analysis using STATA version**-**15.0. The results were summarized in form of bar graphs and tables. The graphs were generated with GraphPad Prism 8.0 software (GraphPad Software, Inc., San Diego, CA).

## Results

### Socio-demographic profiles of participants

All participants were Ugandans with the majority, 59 (74%) being male. Most participants, 36 (45%) were in the age category of 31–40 years while the minority were of ages ≥ 71 and ≤ 30 years. Seventy-nine (99%) of the respondents were married while 1 (1%) was single. Most of the respondents, 49 (62%), had attained the primary level of education while the minority, 1 (1%) were had tertiary education (Table 1).

**Table 1:** Socio-demographic characteristics of herbalists in Butaleja District, Eastern Uganda

Characteristic	Frequency	Percentage (%)
Gender		
Male	59	74
Female	21	26
Age (years)		
10–20	1	1
21–30	8	10
31–40	36	45
41–50	26	33
51–60	4	5
61–70	3	4
≥ 71	2	2
Nationality		
Ugandan	80	100
Non-Ugandan	0	0
Marital status		
Married	79	99
Single	1	1
Education		
Informal education	2	2
Primary level	49	62
Secondary level	28	35
Tertiary education	1	1

### Diversity of medicinal plant species

A total of 133 medicinal plant species belonging to 34 families and 125 genera were identified (Additional file 1: Table S1). The dominant family was Fabaceae with 22 species, followed by Solanaceae and Asteraceae with 10 species each, Euphorbiaceae and Myrtaceae; 6 with 6 species each. The total frequency of mention (TFM) for a particular medicinal plant species ranged between 1 and 98. The ten most frequently mentioned species were *Mangifera indica* L. (TFM = 98), *Momordica foetida* Schumach (TFM = 96), *Aloe wollastonii* Rendle (TFM = 81), *Tamarindus indica* L (TFM = 80), *Cannabis sativa* L. (TFM = 73) *Aristolochia elegans* Mast. (TFM = 72), *Vernonia amygdalina* Delile (TFM = 62), *Bidens pilosa* L. (TFM = 61), *Aloe vera* (L.) Burm.f. (TFM = 59) and *Carica papaya* L. (TFM = 55). The major plant part used in herbal medicine preparations was the leaf; 81 (80%) followed by the root; 15 (15%). Out of the 133 medicinal plant species recorded, herbaceous species were dominant. There was wide variation in the number of diseases treated by each species. For instance, *Persea americana* Miller, *Rumex usambarensis* (Dammer), *Allium sativum* L., *Mangifera Indica* L.*, Aristolochia elegans*, *Aloe vera (L.)* were reported to treat more than four diseases each.

### Modes of administration and diseases treated

Drinking of decoctions, was the commonest method of administration, (34.6%); followed by drinking of infusions, (16%); and the use of concoctions, (8.3%) (Additional file 1: Table S1). The identified plant species were reported to treat 101 physical ailments and spiritual diseases. The dominant ailment treated was cough (7.7%) followed by ulcers (7.4%), malaria (4.5%), candidiasis (3.9%) and stomachache (3.9%) (Table 2). Some medicinal plant species such as *Momordica foetida*, *Tamarindus indica*, *Mangifera indica* and *Aloe wollastonii* had higher frequency of mention for treatment of Bromhidrosis (FM = 55), Blood pressure (FM = 54) cough (FM = 49) and malaria (FM = 42), respectively. There were similarities and variations in the use of plant species in the different villages in the study area. Diseases whose frequency of citation was between 1 and 4 were categorized among “others” of which 44, 25, 9 and 5 had citation of 1, 2, 3 and 4, respectively. Most herbalists believed in spiritualism and witchcraft, and thus some plants, such as *Dyschoriste radicans* (Hochst. ex A. Rich.) Nees, and *Chenopodium ambrosoides* L*.,* were being used in the management of complications related these two beliefs. Such complications included evil spirit possession, madness, swollen legs and bad luck among others (Additional file 1: Table S1).

**Table 2:** Major diseases treated by the identified medicinal plants

Ailment	Frequency of citation	% of citation
Cough	24	7.7
Ulcers	23	7.4
Malaria	14	4.5
Candidiasis	12	3.9
Stomachache	12	3.9
Diarrhea	9	2.9
Erectile dysfunction	9	2.9
Dystocia labor	8	2.6
Gastro-intestinal complications	7	2.3
Evil spirit possession	7	2.3
Cutaneous mycoses	6	1.9
Gonorrhea	6	1.9
Hypertension	6	1.9
Measles	6	1.9
Breech position in pregnancy	5	1.6
Chest pain	5	1.6
Fever	5	1.6
Yellow fever	5	1.6
Others	141	45.5

### Sources of medicinal plants and traditional medicinal knowledge (TMK)

**Fig. 2:**
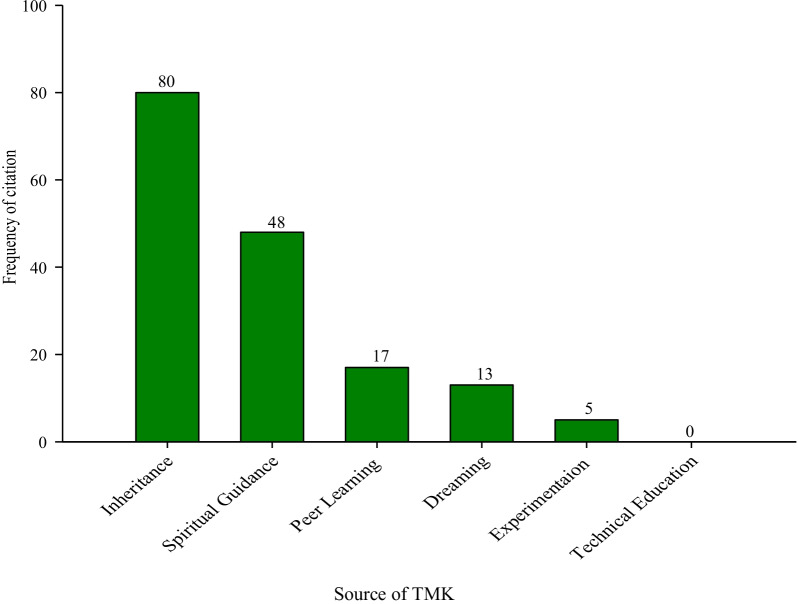
Sources of TMK among traditional medical practitioners in selected rural villages in Butaleja District, Eastern Uganda

Homestead farms and roadside vegetation were cited as the commonest sources of medicinal plants, with each mentioned by all the THP (100%). Others included; distant forests, (10%); markets, (9%); and spiritually protected forests, (Engolo). Wetlands were the rarest source of medicinal plants (6%). All the THP mentioned obtaining TMK as an inheritance from parents and grandparents. Experimentation (random selection and medicinal use of plants species without having prior knowledge of their effectiveness), was the least mentioned source of TMK (6%) (Fig. 2).

### Informant consensus factor

Fifteen disease categories were identified (Table 3). The highest ICF was reported for metabolic disorders (0.952), with five species and 84 use reports. This was followed by cardiovascular diseases (0.932), optical ailments (0.927), and spiritual complications (0.906). Only one disease category had an ICF value of less than 0.800, viz cancers (0.619) (Table 3).

**Table 3:** Informant consensus factor by categories of diseases in Butaleja District

Use category	Ailments	Use citation (N_ur_)	Number of species used (N_t_)	Informant consensus factor (ICF)
Aches	Backache, joint pains, migraine, myalgia, toothache	43	9	0.810
Skin infections	Acne, athlete's foot, cutaneous mycoses, dermatophytosis, lethargy, other skin diseases	130	17	0.877
Cancers	Breast cancer, colon cancer, esophageal cancer, stomach cancer, throat cancer, other cancers	22	9	0.619
Cardiovascular diseases	Anemia, high blood pressure, heart diseases, hypertension	119	9	0.932
Ear, nose and throat	Nose bleeding, septic ears, sore throat, tonsillitis	46	7	0.867
Gastro-intestinal tract infections	Abdominal pains, diarrhea, dysentery, abdominal hernia, indigestion, flatulence, stomachache, ulcers, vomiting, others	320	47	0.856
Bone diseases	Bone fracture, osteodynia, osteomyelitis, osteoporosis, rheumatism, septic arthritis (osteomyelitis)	57	9	0.857
Metabolic disorders	Diabetes, bromhidrosis	84	5	0.952
Urinogenital, and reproductive disorders	Erectile dysfunction, hydrocele, genital warts, syphilis, uterine fibroids, menorrhagia, retained placenta, gonorrhea, abnormal menstrual cycle, infertility, breech position in pregnancy, vaginal candidiasis, dystocia labor	294	42	0.860
Optical complications	Itchy eyes, visual impairment	42	4	0.927
Parasitic diseases	Malaria, stomach helminthiasis	254	17	0.937
Respiratory infections	Asthma, chest congestion, chest pain, cough, flu, pneumonia, whooping cough	287	35	0.881
Spiritual complications	Witchcraft, evil spirit possession, lack of spiritual power, nightmares, spirit possession	139	14	0.906
Viral diseases	Measles, mumps, yellow fever	77	10	0.882
Other purposes	Abscess, allergy, fever, gangrenes, psychotic excitement, snake bite envenomation, colic in babies, hypo-galactorrhea, lack of appetite, insomnia, poor memory	86	17	0.812

### Conservation of medicinal plants and traditional medicinal knowledge

**Fig. 3:**
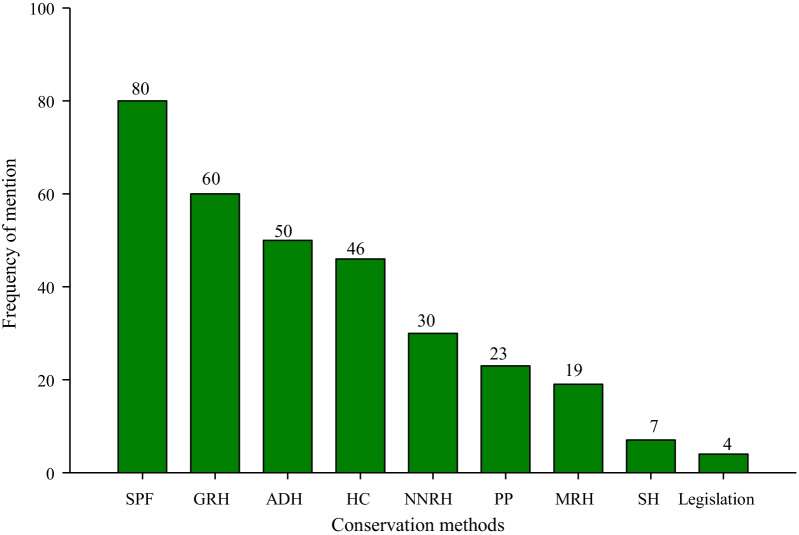
Conservation strategies of medicinal plants among traditional medical practitioners in selected rural villages in Butaleja District, Eastern Uganda. *SPF* spiritually protected forests (Engolo), *GRH* gender restricted harvesting, *ADH* avoiding destructive harvesting, *PP* preservation of propagules, *SH* safeguard from herbivores, *HC* homestead cultivation, *NNRH* night and naked restricted harvesting, and *MRH* mouth restricted harvesting

To ensure sustainability of herbal medicine use, most of the TMP (73%) mentioned that they made efforts to conserve medicinal plants compared to 21 (27%) did not besides abiding to the communal restrictions. Preservation of spiritually protected forests, locally known as “Engolo” was the most frequently mentioned conservation strategy, 80 (100%); while compliance with government laws and policies was the least mentioned approach, 4 (5%) (Fig. 3).

## Discussion

### Demographic characteristics

The majority of the participants in this study were males. The dominance of male herbalists was also reported earlier in other parts of Africa [29, 30]. On the contrary, an earlier study in South Africa reported female herbalists to be more dominant than males [31]. The dominance of male traditional medical practitioners is in line with the African belief, that traditional healers should mainly be male [32–34]. In the present study, close to 50% of the participants were in the active age category of 31–45 years. This is commendable and shows that the herbal medicine sector in Butaleja has the potential for future continuity and expansion. The young population will mitigate the risk of TMK loss by passing it on to future generations. This finding contradicts those of a similar study in communities adjacent to Mabira Central Forest Reserve (CFR) in Uganda where herbal medicine practice was dominated by the elderly [35]. In Butaleja, the predominance of the youths in the herbal medicine sector in Butaleja could partly be attributed to high unemplyment rates. This leaves the sector as a lucrative alternative source of income for the young generation.

### The diversity of medicinal plant species

There was a high diversity of medicinal plants used by residents of Butaleja district to treat diseases including: cancers, cardiovascular, gastro-intestinal, urinogenital, and reproductive disorders. This is an indication of the significant role herbal medicine plays in meeting the basic healthcare needs in the area given limited health facilities. The medicinal plant species used belong to Fabaceae, Solanaceae, Asteraceae, and Euphorbiaceae. These families were also reported in earlier studies, to have high numbers of medicinal plants [35–39]. This could possibly be attributed to their higher diversity and abundance in the study area. In agreement with earlier studies [40–45] the leaf was the most frequently used organ, followed by the roots. However, this is in contradiction with the findings of [39], who reported roots as the most preferred organ for use as medicine in rural communities. The popularity of the leaves in herbal medicine utilization in Uganda was also reported in earlier studies [14, 38, 46]. This was attributed to their easy accessibility [47] and harvesting [14].

In the current study, we noted a common perception of the root as a more potent organ than any other plant organ. This partly accounts for its notable use as the second most preferred organ. In conformity with this perception, higher use of roots has been associated with their high partitioning for the photosynthates or exudates [48], which act as toxins for protection against devourers; and in most cases, these compounds are of medicinal value to the humans [49]. However, the frequent use of roots threatens the survival of the medicinal plants as compared to the leaves, which usually regenerate under favorable conditions to perpetuate the occurrence of the medicinal plant.

### Ailments treated and modes of preparation and administration

The various diseases treated using medicinal plants in the study area could be attributed to the trust local communities attach to herbal medicine. This number is higher than that reported in some earlier studies carried out in rural communities of Uganda [40, 41] and [50] in Eastern Madagascar. The use of herbal medicines to treat and manage both common ailments, such as cough, malaria, candidiasis, stomachache and diarrhea as well as more specialized complications such breach position in pregnancy, dystocia labor, diabetes, cancer, and stomach ulcer is a clear indicator of the importance of herbal medicine to residents of Butaleja. In line with earlier observation [51], the over reliance on medicinal plants in the area could be attributed to their availability and affordability. This could also be attributed to inadequate access to modern medicine [50], evidenced by presence of one Health Centre IV hospital serving the highly population in the three study villages.

The dominant use of decoction in herbal medicine preparation followed by infusion and concoction is not limited to the current study area. Previous studies carried out in the rural communities of Uganda and other parts of the world [14] and [52] revealed a similar trend. Decoction involves boiling, which enhances extraction of active ingredients from medicinal plants, hence enhancing medicinal efficacy, however, if not done cautiously, it may cause degradation of metabolites [53]. Boiling also preserves the herbal remedies for a longer period compared to cold extraction.

### Sources of medicinal plant species and TMK

Traditionally, medicinal plant materials are mainly collected from the wild [15]. Contrary to the general trend, medicinal plant species in the current study were majorly collected from homestead-farms. This could be attributed to the widespread destruction of wild habitats especially wetlands, through cultivation of paddy rice; population explosion due to early marriages and high school drop-outs rates; and the burgeoning commercialization of herbal medicine that has led to over-exploitation of wild populations. Over-exploitation of medicinal plant species was reported earlier [54], as one of the major driving factors for medicinal plant species scarcity in communities. The study revealed that acquisition and conservation of TMK in this area is mainly attained through inheritance from elders, both orally and through practice. This practice has also been reported to be predominant in other rural communities in Uganda [55] and other countries in Africa [56]. Erosion of medicinal plant diversity and TMK is likely to be exacerbated by loss of medicinal plant habitats through wetland cultivation of rice, rural–urban migration of the youth, cultivation of rice in wetlands, population pressure and lack of concerted effort to conserve the environment.

### The informant consensus factor

With the exception of cancers which had an ICF of 0.619, the value was high for all the other disease categories (≥ 0.800). The high ICF values mean that there is a high level of agreement regarding the plant species used to treat various ailment categories in the study area and that such species are effective [57]. The fact that the ICF values were high reflects a great diversity of plant species used in the management of human diseases in the study community [28]. The low ICF for cancers may be attributed to lack of equipment for cancer diagnosis in the area and the fact that its symptoms such as vomiting, nausea, diarrhea, fatigue and headache [58] are also symptoms of other ailments that were commonly cited such as gastro-intestinal complications and cardiovascular infection. Consequently, cancers might be misperceived as other common ailments. Our findings are in agreement with those of [27], who reported exceptionally high ICF values for plants used in the herbalist communities of Luocheng Mulam county in China. The high values of ICF observed could be attributed to: the use of the same species by a large proportion of informants, and the presence of a variety of community practices (Fig. 2) aimed at sharing TMK.

On the other hand, the low ICF values observed in the current study may possibly be attributed to, (1) the fact that herbalists are often secretive with their traditional medicinal knowledge leading to differences in the species they cite for a particular disease category; (2) availability of a rich diversity of medicinal plants species; and (3) differences in the sources of medicinal traditional knowledge in herbalists’ communities.

### Conservation of medicinal plants and traditional medicinal knowledge

Though the conservation of medicinal plants was highly threatened by multiple factors such as habitat loss, population pressure and the increasing dependence on herbal medicine as a source of income, their conservation was promoted through cultural beliefs and norms. These included; gender-restricted harvesting, mouth harvesting, night and naked harvesting, and the gazetting of spiritually protected forests (Engolo) for each clan, from which harvesting is only authorized by the clan head. Further, there was a common belief among many herbalists that the onus to conserve medicinal plants and the associated knowledge was a divine duty entrusted on them by ancestral spirits. Spiritual beliefs have been reported to play a major role in conservation of resources [59, 60]. In addition, the predominant use of leaves as a preferred organ, contributes to sustainable utilization of medicinal plants, since in most cases, it eliminates destructive harvesting. The study revealed a high traditional medicinal knowledge transfer through spiritualism and dreaming, which are not very much recognized in traditional medicine knowledge transfer and conservation.

Though peer learning was also cited, it is likely to become minimal with increased commercialization of the herbal medicine industry, due to fear of competition among the herbalists. Notably missing was the adoption of technical training of the young generation as a means of conserving TMK. This points to complete lack of integration of herbal medicinal use into the health care system. Therefore, there is no reliable method of indigenous medicinal knowledge conservation in the study community. This calls for the need to devise reliable conservation measures to arrest the possible loss of TMK in the rural communities as previously reported elsewhere [45]. Compliance with government laws and policies was the least mentioned strategy adopted by the herbalists in an attempt to promote conservation of medicinal plants. This might partly be attributed to ignorance about the laws, and/or lack of concerted efforts by the government to enforce the regulations that would safeguard medicinal plant diversity conservation.

## Conclusion and recommendations

This study showed that Butaleja district has a high diversity of plant species used as remedies for several ailments. The high dependency on herbal medicine for primary healthcare and income generation in Butaleja district calls for preservation of this resource and the associated TMK. A majority of the medicinal plant species used belong to the Fabaceae and Solanaceae, and they were most frequently used in managing some of the world’s most killer diseases, such as; respiratory tract ailments and malaria. There was a general consensus on the plant species used to manage diseases in different categories which is a clear indicator that such plants are effective and worth further investigation for bioactive ingredients and their associated pharmacological activities. This will be instrumental in establishing the dosages, toxicity and efficacy levels of different herbal preparations and could act as a springboard to discovery on new plant-based drugs.

The conservation of medicinal plants, and the associated indigenous traditional knowledge, was strongly attributed to traditional cultural beliefs and norms. Therefore, traditional cultural values should be incorporated into the national biodiversity conservation agenda, owing to the extraordinary role of culture in inspiring the conservation of medicinal plants and the TMK observed in this study. There is need for laboratory-based experiments on the plants identified in this study to confirm their safety and efficacy. This would support the adoption of these plants in the global fight against the deadly diseases they were claimed to treat.

## Supplementary Information

**Additional file 1. Table S1.** Medicinal plants used for treatment of common ailments in Butalejja District, Eastern Uganda.

## Data Availability

Data sets generated and analyzed during this study are available from the corresponding author on reasonable request.

## Abbreviations

UNCTS: Uganda national council for science and technology
TMK: Traditional medicinal knowledge
ICF: Informant consensus factor
COVID-19: Corona virus disease- 2019
