# Supplementary material for: Medicinal plant use, conservation, and the associated traditional knowledge in rural communities in Eastern Uganda
Source: Trop Med Health. 2022 Jun 6;50:39. doi: 10.1186/s41182-022-00428-1 (PMC9168352; doi:10.1186/s41182-022-00428-1)
Supplement: Supplementary file 1 — Additional file 1. Table S1. Medicinal plants used for treatment of common ailments in Butalejja District, Eastern Uganda. [file 41182_2022_428_MOESM1_ESM.docx]

**Table S1: Medicinal plants used for treatment of common ailments in Butalejja District, Eastern Uganda**

| Family, scientific name,  (Voucher number), TFM | Local name | FM | HB | Part used | Ailment | Mode of administration |
| --- | --- | --- | --- | --- | --- | --- |
| **Acanthaceae** |  |  |  |  |  |  |
| *Asystasia schimperi* T.Anders.,  (BTH020), 4 | Nanderanghombe (Lun) | 4 | H | Leaf | Stomach helminthiasis | Decoction drunk |
| *Acanthus pubescens* (Thomson ex Oliv.) Engl., (BTH013), 1 | Itovu (Lun), Amatovu (Lug) | 1 | S | Root | Bladder infections | Concoction drunk |
| *Dyschoriste radicans* (A. Rich.) Nees (BTH009), 4 |  | 3 | H | Leaf | Malaria | Infusion bathed |
|  |  | 1 |  | WP | Evil spirits possession | Burnt and smoke inhaled; infusion bathed |
| **Alliaceae** |  |  |  |  |  |  |
| *Allium cepa* L., (BTH039), 10 | Hatungulu (Lun) | 7 | H | Leaf | Cough | Decoction drunk |
|  |  | 3 |  | Bulb | Ulcers | Infusion drunk |
| *Allium sativum* L. (BTH001), 42 | Hatungulucumu (Lun) | 26 | H | Bulb | Chest congestion | Cloves chewed |
|  |  | 3 |  | Bulb | Abscess | Poultice applied on the skin |
|  |  | 4 |  | Bulb | Warts | Poultice applied on the skin |
|  |  | 1 |  | Bulb | Athlete’s foot | Fresh garlic juice applied on the affected part |
|  |  | 1 |  | Bulb | Teething babies | Rub the gum with the clove poultice |
|  |  | 7 |  | Bulb | Rheumatism | Poultice applied on the painful part of the body |
| **Amaranthaceae** |  |  |  |  |  |  |
| *Chenopodium ambrosoides* L.*,* (BTH011), 14 | Katadogo (Lug) | 3 | H | Shoot | Nightmares | Extract smeared on the head at bedtime |
|  |  | 1 |  | Shoot | Spirits | Burn and inhale the smoke |
|  |  | 9 |  | Leaf | Psychotic excitement | Burn and inhale the smoke |
|  |  | 1 |  | Leaf | Stomach gas | Infusion drunk |
| *Chenopodium opulifolium* Koch. & Ziz, (BTH012), 24 | Olugahagohe (Lun) | 6 | H | Leaf | Gangrenes | Poultice applied topically |
|  |  | 18 |  | Leaf | Sore throat | Chew and swallow extract |
| *Gomphrena globosa* L., (BTH064), 2 | Natasuba (Lun) | 2 | H | Shoot | Ulcers | Decoction drunk |
| **Anacardiaceae** |  |  |  |  |  |  |
| *Mangifera indica* L., (BTH090), 98 | Omuyembe (Lug) | 9 | T | Stem bark | Ulcers | Decoction drunk |
|  |  | 34 |  | Leaf | Malaria | Steam bath |
|  |  | 4 |  | Leaf | Diabetes | Decoction drunk |
|  |  | 2 |  | Leaf | Breast cancer | Decoction drunk |
|  |  | 49 |  | Leaf | Cough | Concoction drunk |
| **Apiaceae** |  |  |  |  |  |  |
| *Centella asiatica* (L.) Urban, (BTH024), 16 | Kutukumu (Lun) | 8 | H | Leaf | Poor memory | Decoction drunk |
|  |  | 2 |  | WP | Fever | Decoction drunk |
|  |  | 6 |  | WP | Gastro-intestinal complications | Decoction drunk |

**Table. S1 continued**

| Family, scientific name,  Voucher number), TFM | Local Name | FM | HB | Part used | Ailment | Mode of administration |
| --- | --- | --- | --- | --- | --- | --- |
| **Apocynaceae** |  |  |  |  |  |  |
| *Carissa*edulis (Forssk.) Vahl,  (BTH033), 4 | Omutooga (Lun) | 4 | S | Root | Muscle aches (myalgia) | Decoction drunk |
| *Mondia whitei* (Hook.f.) Skeels, (BTH061), 26 | Mulondo (Lun) | 7 | H | Root | Stomachache | Chew and swallow extract |
|  |  | 29 |  | Root | Erectile dysfunction | Chew and swallow extract |
|  |  | 8 |  | Root | Erectile dysfunction | Infusion drink |
| *Catharanthus roseus* (L.) G.Don. (BTH002), 38 | Kamuli (Lun) | 5 | H | Leaf | Ulcers | Decoction drunk |
|  |  | 5 |  | Leaf | Cancer | Infusion drunk |
|  |  | 19 |  | Leaf | Blood pressure; | Infusion drunk |
|  |  | 9 |  | Shoot | Abnormal menstrual cycle | Decoction drunk |
| *Thevetia peruviana*(Pers.) K.Schum*.,* (BTH051), 1 | Busitani (Lug) | 1 | S | Root | Snake bite | Infusion drunk |
| **Arecaceae** |  |  |  |  |  |  |
| *Phoenix reclinata* Jacq., (BTH065), 1 | Ehyihindu (Lun) | 1 | T | Seed | Erectile dysfunction | Decoction drunk |
| **Aristolochiaceae** |  |  |  |  |  |  |
| *Aristolochia elegans* Mast. (BTH016), 72 | Nakasero (Lug) | 9 | H | Seed | Pneumonia | Infusion drunk |
|  |  | 24 |  | Root | Stomachache | Decoction drunk |
|  |  | 35 |  | Seed | Malaria | Infusion drunk |
|  |  | 2 |  | Seed | Cough | Infusion drunk |
|  |  | 1 |  | Leaf | Bromhidrosis | Concoction applied topically |
|  |  | 1 |  | Leaf | Migraine | Infusion dropped in nostrils |
| **Asphodelaceae** |  |  |  |  |  |  |
| *Aloe vera* (L.) Burm.f. (BTH059), 59 | Ehikaka (Lun) | 35 | H | Leaf | Malaria | Decoction drunk |
|  |  | 8 |  | Leaf | Stomachache | Infusion drunk |
|  |  | 12 |  | Leaf | Acne | Extract topically applied |
|  |  | 4 |  | Leaf | Ulcers | Decoction drunk |
| *Aloe wollastonii* Rendle (BTH010), 81 | Ehikaka (Lun) | 5 | H | Leaf | Yellow fever | Decoction drunk |
|  |  | 7 |  | Leaf | Ulcers | Decoction drunk |
|  |  | 8 |  | Leaf | Acne | Extract topically applied |
|  |  | 42 |  | Leaf | Malaria | Decoction drunk |
|  |  | 8 |  | Leaf | Gastro-intestinal complications | Infusion drunk |
|  |  | 2 |  | Leaf | Snake bite | Infusion drunk |
|  |  | 2 |  | Leaf | Stomachache | Infusion drunk |
|  |  | 2 |  | Leaf | Gonorrhea | Decoction drunk |
|  |  | 3 |  | Leaf | Cough | Concoction drunk |
|  |  | 2 |  | Leaf | Flu | Concoction drunk |

**Table. S1 continued**

| Family, scientific name,  (Voucher number), TFM | Local name | FM | HB | Part used | Ailment | Mode of administration |
| --- | --- | --- | --- | --- | --- | --- |
| **Asteraceae** |  |  |  |  |  |  |
| *Bidens pilosa* L., (BTH032), 64 | Obuhaala (Lun) | 9 | H | Leaf | Itchy eyes | Infusion dropped in eyes |
|  |  | 18 |  | Leaf | Ulcers | Decoction drunk |
|  |  | 30 |  | Leaf | Wounds | Extract applied topically |
|  |  | 7 |  | Leaf | Ulcers | Steamed leaves eaten |
| *Conyza sumatrensis* (Retz.) E.H. Walker, (BTH014), 14 | Kafumbe omusajja (Lug) | 7 | H | Leaf | Itchy eyes | Infusion dropped in eyes |
|  |  | 1 |  | Leaf | Blood pressure | Decoction drunk |
|  |  | 6 |  | Leaf | Yellow fever | Infusion drunk |
| *Melanthera scandens* (Schuach & Thonn.) Roberty, (BTH008), 1 | Makaayi (Lug) | 1 | H | Leaf | Chest pain | Infusion drunk |
| *Microglossa angolensis* Oliv. & Hiern., (BTH045), 3 | Akalulusa ahasinde (Lun) | 3 | H | Root  Leaf | Dysentery | Decoction drunk |
| *Microglossa pyrifolia* (Lam.) O.Kuntze, (BTH019), 3 |  | 3 | S | Leaf | Diarrhea | Decoction drunk |
| *Schkuhria pinnata*(Lam.) Thell, (BTH030), 19 |  | 10 | H | Leaf | Stomachaches | Infusion drunk |
|  |  | 9 |  | Shoot | Fever | Infusion drunk |
| *Sonchus asper* (L.) Hill. (BTH038), 9 |  | 9 | H | Leaf | Tonsillitis | Infusion drunk |
| *Synedrella nodiflora* (L.) Gaertn., (BTH047), 5 |  | 4 | H | Shoot | Evil spirit possession | Infusion drunk |
|  |  | 1 |  | Root | Cough | Decoction drunk |
| *Seriphium kilimandscharicum* (O.Hoffm.) Koekmoer, (BTH028), 7 | Omututu (Lun) | 7 | S | Root | Mouth sores | Powder applied on sores |
| *Tagetes minuta*L*.,* (BTH029), 12 | Kawunyira (Lug) | 12 | H | Leaf | Evil spirit possession | Smoke inhaled |
| *Vernonia amygdalina* Delile., (BTH021), 62 | Omululusa (Lun) | 33 | S | Leaf | Malaria | Infusion drunk |
|  |  | 5 |  | Leaf | Ulcers | Decoction drunk |
|  |  | 8 |  | Leaf | Malaria | Concoction drunk |
|  |  | 8 |  | Root | Lack of appetite | Decoction drunk |
|  |  | 8 |  | Root | Stomach helminthiasis | Decoction drunk |
| *Vernonia cinerea* (L.) Less. (BTH042), 6 | Kayayana (Lug) | 6 | H | Leaf | Stomach helminthiasis | Decoction drunk |
| **Basellaceae** |  |  |  |  |  |  |
| *Basella alba* L., (BTH007), 3 |  | 3 | H | Leaf | Dystocia labor | Infusion drunk and bathed |
| **Bignoniaceae** |  |  |  |  |  |  |
| *Kigelia africana* (Lam.) Benth., (BTH003), 19 | Omujungwe (Lun) | 5 | T | Root bark | Urinary Tract Obstruction | Decoction drunk |
|  |  | 8 |  | Stem bark Flower | Pressure | Decoction drunk |
|  |  | 1 |  | Stem bark | Syphilis | Decoction drunk |
|  |  | 5 |  | Seed | Pressure | Decoction drunk |
| *Spathodea campanulata*P.Beauv.  (BTH016), 4 | Omudungumuliro (Lun) | 4 | T | Stem | Candidiasis | Decoction drunk |

**Table. S1 continued**

| Family, scientific name,  (Voucher number), TFM | Local name | FM | HB | Part used | Ailment | Mode of administration |
| --- | --- | --- | --- | --- | --- | --- |
| *Markhamia lutea* (Benth.) K. Schum., (BTH005), 40 | Omusoolwa (Lun), Musambya (Lug) | 13 | S | Root | Dystocia labor | Ash licked |
|  |  | 10 |  | Stem bark | Swollen legs due to witchcraft | Crush and smear on skin cuts |
|  |  | 14 |  | Flower | Septic ears | Extract dropped into ears |
|  |  | 1 |  | Root | Osteoporosis | Decoction drunk |
|  |  | 2 |  | Root | Menorrhagia | Concoction drunk |
| **Brassicaceae** |  |  |  |  |  |  |
| *Brassica oleracea* L. (BTH022), 9 |  | 3 | H | Root | Dystocia Labor | Root chewed and extract swallowed |
|  |  | 6 |  | Leaf | Ulcers | Infusion drunk |
| **Bromeliaceae** |  |  |  |  |  |  |
| *Ananas comosus* (L.) Merril, (BTH027), 5 | Nanansi (Lug) | 1 | H | Fruit | Latent infections | Fruit eaten |
|  |  | 3 |  | Fruit | Oesophageal cancer | Concoction drunk |
|  |  | 1 |  | Leaf | Joint pains | Decoction drunk |
| **Celestraceae** |  |  |  |  |  |  |
| Maytenus senegalensis (Lam.) Exell (BTH031), 2 |  | 2 | T | Stem | Syphilis | Decoction drunk |
| **Canellaceae** |  |  |  |  |  |  |
| *Warburgia ugandensis* Sprague, (BTH006), 31 |  | 24 | T | Stem bark | Gastro-intestinal complications | Infusion of powder drunk |
|  |  | 4 |  | Leaf | Whooping cough | Decoction drunk |
|  |  | 3 |  | Stem bark | Candidiasis | Decoction drunk |
| **Cannabaceae** |  |  |  |  |  |  |
| *Cannabis sativa* L. (BTH017), 73 | Njaga (Lug, Lun) | 22 | H | Leaf | Rheumatism | Infusion drunk |
|  |  | 10 |  | Leaf | Measles | Decoction drunk |
|  |  | 41 |  | Leaf | Lethargy | Decoction drunk |
| **Capparaceae** |  |  |  |  |  |  |
| *Boscia* *coriacea* Graells (BTH026), 4 | Endebera (Lun), | 4 | S | Root | Stomachache | Decoction drunk |
| *Capparis tomentosa* Lam. (BTH004), 8 | Muzingaanyi (Lun) | 8 | S | Root | Gastro Intestinal complications | Infusion of powder drunk |
|  |  |  |  | Root | Evil spirit possession | Smoked or powder bathed |
| *Capparis sepiaria*L***.*** (BTH018), 9 | Omutasubwa (Lun) | 9 | S | Root | Erectile dysfunction | Decoction drunk |
| *Cleome gynandra* L. (BTH050), 28 | Essaaga (Lun) | 8 | H | Root | Dystocia labor | Root chewed and extract swallowed |
|  |  | 12 |  | Flower | Dermatophytosis | Poultice applied topically |
|  |  | 2 |  | Leaf | Hernia | Infusion drunk |
|  |  | 2 |  | Leaf | Ulcers | Infusion drunk |
|  |  | 4 |  | Root | Dermatophytosis | Poultice mixed with paraffin and applied topically |

**Table. S1 continued**

| Family, scientific name,  (Voucher number), TFM | Local name | FM | HB | Part used | Ailment | Mode of administration |
| --- | --- | --- | --- | --- | --- | --- |
| **Caricaceae** |  |  |  |  |  |  |
| *Carica papaya* L. (BTH025), 55 | Omupapaali (Lun) | 25 | S | Fruit | Wounds | Powder applied topically |
|  |  | 19 |  | Seed | Stomach helminthiasis | Seed swallowed |
|  |  | 7 |  | Seed | Urinary tract infections | Fresh Seed chewed and extract swallowed |
|  |  | 4 |  | Leaf | Anemia | Infusion drunk |
| **Convolvulaceae** |  |  |  |  |  |  |
| *Ipomoea batatas* (L.) Lam., (BTH062), 1 | Lumonde (Lug), Amabooni (Lun) | 1 | H | Tuber | Diarrhea | Decoction of crashed peeled tuber drunk |
| **Cucurbitaceae** |  |  |  |  |  |  |
| *Kedrostis foetidissima* (Jacq.) Cogn., (BTH054), 8 | Nasiniebi (Lun) | 3 | H | Leaf | Lack of appetite | Powder mixed with food and eaten |
|  |  | 5 |  | Leaf | Measles | Decoction drunk |
| *Luffa cylindrica* (L.) M.J. Roem. (BTH034), 17 | Ekyangwe (Lug) | 17 | H | Root | Dystocia labor | Root chewed |
| *Momordica foetida* Schumach, (BTH058), 96 | Amabwombe (Lun) | 55 | H | Leaf | Bromhidrosis | Infusion bathed |
|  |  | 41 |  | Leaf | Cough | Infusion drunk |
| **Dracaenaceae** |  |  |  |  |  |  |
| *Zehneria minutiflora* (Cogn.) C. Jeffrey, (BTH040), 8 | Nambula kifo (Lun) | 8 | H | WP | Witchcraft | Place in footsteps of victim |
| *Dracaena fragrans* (L.) Ker- Gawl.  (BTH055), 17 | Namuhirangwengwe (Lun) | 17 | S | Stem | Lack of spiritual power | Stem with its leaf held by subject |
| *Dracaena steudneri* Engl.,( BTH060), 7 |  | 7 | T | Stem bark | Abdominal pains | Decoction drunk |
| **Ebenaceae** |  |  |  |  |  |  |
| *Euclea schimperi*(A.DC.) Dandy,  (BTH041), 2 | Mangholu (Lun) | 2 | S |  | Ulcers | Decoction drunk before meals. |
| **Euphorbiaceae** |  |  |  |  |  |  |
| *Euphorbia heterochroma* Pax, (BTH057), 20 | Ahadunga (Lun) | 5 | S | Stem bark | Diarrhea | Decoction drunk |
|  |  | 15 |  |  | Evil spirit possession | Planted in a homestead |
| *Euphorbia hirta* Lnn., (BTH035), 10 | Akasandasanda (Lug) | 5 | H | Stem | Migraine | Stem sap dropped into nostrils |
|  |  | 2 |  | Leaf | Asthma | Decoction drunk |
|  |  | 3 |  | WP | Urinary tract infections | Decoction drunk |
| *Flueggea virosa* (Willd.) Voigt, (BTH052), 25 | Oluhandwa (Lun) | 4 | S | Root | Cough | Decoction drunk |
|  |  | 21 |  | Root | Candidiasis | Concoction drunk |

**Table. S1 continued**

| Family, scientific name,  (Voucher number), TFM | Local name | FM | HB | Part used | Ailment | Mode of administration |
| --- | --- | --- | --- | --- | --- | --- |
| *Jatropha multifida* L., (BTH056), 3 |  | 3 | H | Root | Edema | Crush and smear after skin cuts |
| *Senna occidentalis* (L.) Link, (BTH073), 6 | Sagalasaasi (Lun) | 6 | S | Root and Leaf | Stomachache | Infusion drunk |
| *Sesbania sesban* (L.) Merril, (BTH049), 11 | Lusabasabi (Lun) | 11 | S | Leaf | Stomachache | Infusion drunk |
| *Tamarindus indica* L., (BTH066), 80 | Omukooge (Lun) | 7 | T | Fruit | Constipation | Infusion drunk |
|  |  | 19 |  | Stem bark | Uterine fibroids | Decoction drunk |
|  |  | 54 |  | Fruit | Blood pressure | Decoction drunk |
| *Tylosema fassoglensis* (Kotschy ex Schweinf.) Torre & Hillc. (BTH070), 9 | Ahayuge (Lun) | 5 | S | Tuber | Diarrhea | Decoction drunk |
|  |  | 4 |  | Tuber | Candidiasis | Concoction drunk |
| **Flacourtiaceae** |  |  |  |  |  |  |
| *Dovyalis macrocalyx* (Oliv.) Warb. (BTH063), 4 | Sundo mumwa (Lun) | 4 | T | Root | Osteoporosis | Decoction drunk |
| **Guttiferae** |  |  |  |  |  |  |
| *Psorospermum febrifugum* Spach, (BTH046), 15 | Akanzironziro (Lug) | 6 | T | Root | Fibroids | Decoction drunk |
|  |  | 9 |  | Stem bark | Skin diseases | Powder mixed with Vaseline and topically applied |
| **Lamiaceae** |  |  |  |  |  |  |
| *Hoslundia opposita* Vahl, (BTH072), 28 | Efoodo (Lun) | 7 | S | Root | Yellow fever | Decoction drunk |
|  |  | 8 |  | Leaf | Ulcers | Decoction drunk |
|  |  | 13 |  | Leaf | Wounds | Decoction drunk |
| *Leonotis nepetifolia* (L.) Ait.f., (BTH053), 29 | Ehifumufumu (Lun) | 18 | H | Leaf | Gastro-intestinal complications | Decoction drunk |
|  |  | 11 |  | Leaf | Chest pain | Infusion drunk |
| *Leucas martinicensis* (Jacq.) R. Br., (BTH048), 8 | Nagasusuuni (Lun) | 8 | H | Leaf | Migraine | Drops in nostrils |
| *Ocimum gratissimum* L., (BTH071), 38 | Omujaaja (Lug) | 15 | H | Leaf | Ulcers | Decoction drunk |
|  |  | 6 |  | Root | Yellow fever | Concoction drunk. |
|  |  | 1 |  | Leaf | Lethargy | Decoction drunk |
|  |  | 12 |  | Leaf | Diarrhea | Decoction of powder drunk |
|  |  | 4 |  | Stem bark | Candidiasis | Decoction drunk |
| *Tetradenia riparia* (Hochst.) Codd,(BTH069), 24 | Ehiyongobela (Lun) | 5 | S | Leaf | Dysentery | Bake leaf wrapped in a banana leaf in hot ash, squeeze out juice and drink |
|  |  | 6 |  | Leaf | Candidiasis | Decoction drunk |
|  |  | 13 |  | Leaf | Cutaneous mycoses | Burnt for a few minutes and applied topically |
| **Lauraceae** |  |  |  |  |  |  |
| *Persea americana* Miller, (BTH067), 37 | Fekedo (Lun) | 3 | T | Seed | Yellow fever | Sees sliced, air dried and decoction of powder drunk as herbal tea |

**Table. S1 continued**

| Family, scientific name,  (Voucher number), TFM | Local name | FM | HB | Part used | Ailment | Mode of administration |
| --- | --- | --- | --- | --- | --- | --- |
|  |  | 1 |  | Leaf | Throat cancer | Decoction drunk |
|  |  | 2 |  | Leaf | Breast cancer | Decoction drunk |
|  |  | 3 |  | Leaf | Malaria | Steam bath/ Decoction drunk |
|  |  | 9 |  | Leaf | Cough | Decoction drunk |
|  |  | 4 |  | Leaf | Anemia | Decoction drunk |
|  |  | 5 |  | Seed | Blood pressure | Decoction of the powder drunk |
|  |  | 3 |  | Leaf | Chest pain | Decoction drunk |
|  |  | 3 |  | Seed | Gonorrhea | Decoction drunk |
|  |  | 2 |  | Root | Gonorrhea | Decoction drunk |
|  |  | 2 |  | Root | Stomachache | Root baked and chewed |
| **Lythraceae** |  |  |  |  |  |  |
| *Punica granatum* Linn, (BTH080), 10 | Nkomamawanga (Lug) | 6 | T | Fruit | Urinary tract infection | Decoction drunk |
|  |  | 4 |  | Fruit | Cough | Decoction drunk |
| **Malvaceae** |  |  |  |  |  |  |
| *Abelmoschus esculentus*(L.) Moench. (BTH068), 11 |  | 7 | S | Leaf | Ulcers | Decoction of the, powder taken as herbal tea |
|  |  | 3 |  | Fruit | Stomach cancer | Eaten as food/spice |
|  |  | 1 |  | Fruit | Colon cancer | Eaten as food/spice |
| *Corchorus olitorius* L. (BTH044), 2 | Mutele (Lug) | 2 | H | WP | Warts | Squeeze and apply poultice topically |
| *Hibiscus acetocella* (BTH043), 7 | Musayi (Lug) | 7 | S | Leaf | Anemia | Decoction drunk |
| **Meliaceae** |  |  |  |  |  |  |
| *Azadirachta indica* A. Juss. (BTH037), 12 |  | 7 | T | Leaf | Malaria | Decoction drunk |
|  |  | 5 |  | Leaf | Cough | Decoction drunk |
| **Menispermaceae** |  |  |  |  |  |  |
| *Cissampelos mucronata* A. Rich. (BTH086), 27 | Nvamagombe (Lun) | 3 | H | Root | Erectile dysfunction | Root chewed and extracts swallowed |
|  |  | 5 |  | Root | Stomachache | Concoction drunk |
|  |  | 4 |  | Stem | Breech position in pregnancy | Stem tied around waist |
|  |  | 8 |  | Leaf | Miscarriages | Infusion drunk |
|  |  | 7 |  | Root | Septic arthritis (Osteomyeritis) | Infusion bathed |
| **Moraceae** |  |  |  |  |  |  |
| *Artocarpus heterophyllus* Lam*.* (BTH074), 3 | Ffene (Lun) | 3 | T | Seed | Ulcers | Concoction drunk |
| *Ficus natalensis* Hochst., (BTH036), 9 | Omutuba (Lug) | 4 | T | Leaf | Malaria | Steam bath |
|  |  | 5 |  | Leaf | Nose bleeding | Inhale smoke from the burnt leaves |
| *Milicia* *excelsa* (Welw.) C.C.Berg, (BTH103), 6 | Omughii (Lun) | 4 | T | Root | Measles | Boil in milk and drink |
|  |  | 2 |  | Root bark | Cutaneous mycoses | Mix powder with Vaseline and apply topically |

**Table. S1 continued**

| Family, scientific name,  (Voucher number), TFM | Local name | FM | HB | Part used | Ailment | Mode of administration |
| --- | --- | --- | --- | --- | --- | --- |
| **Musaceae** |  |  |  |  |  |  |
| *Musa ^x^ paradisiaca* var*. sapientum*  (BTH081), 4 | Amatooke (Lug) | 4 | H | Peels | Cutaneous mycoses | Extract applied topically |
| *Musa ^x^ paradisiaca* var*. paradisiaca,* (BTH096), 9 | Ndizi (Lug) | 9 | H | Inflorescence | Pressure | Air dried powder mixed with food and eaten or decoction of powder taken as herbal tea |
| *Musa sapientum* Linn., (BTH100), 4 | Kisubi (Lun) | 1 | H |  | Breech position in pregnancy |  |
|  |  | 3 |  | Fruit | Ulcers | Peel’s ash leaked |
| **Myrtaceae** |  |  |  |  |  |  |
| *Callistemon citrinus*(Curtis) Skeels (BTH091), 23 | Mwambala butonya (Lug) | 14 | S | L | Cough | Concoction drunk |
|  |  | 9 |  | Stem bark | Cough | Ash licked |
| *Corymbia citriodora* (Hook.) K.D.Hill & L.A.S.Johnson (BTH075), 17 | Kalitunsi (Lug) | 17 | T | Leaf  Stem bark | Cough | Concoction drunk |
| *Eucalyptus globulus*Labill., (BTH010), 11 | Kalitunsi (Lug) | 11 | T | Leaf  Stem bark | Cough | Decoction drunk |
| *Eucalyptus* *grandis* W.Hill, (BTH082), 8 | Kalitunsi (Lug) | 8 | T | Stem bark | Toothache | Powder applied on teeth |
| *Eucalyptus saligna*Sm**.** (BTH092), 8 | Kalitunsi (Lug) | 6 | T | Leaf | Chest pain | Decoction drunk |
|  |  | 2 |  | Leaf | Candidiasis | Concoction drunk |
| *Psidium guajava* L., (BTH101), 18 | Epeera (Lug) | 12 | T | Leaf | Diarrhoea | Decoction drunk |
|  |  | 6 |  | Leaf | Cough | Concoction drunk |
| **Oleaceae** |  |  |  |  |  |  |
| *Jasminium eminii* Gilg. (BTH089), 4 | Kafulu (Lun) | 4 | H | Leaf | Tonsillitis | Ash licked |
| **Passifloraceae** |  |  |  |  |  |  |
| *Adenia cissampeloides* (Hook.) Harms. (BTH083), 5 | Gerogero (Lun) | 5 | H | Leaf | Heart diseases | Decoction drunk |
| *Passiflora edulis* Sims, (BTH109), 3 | Akatunda Lug) | 3 | H | Leaf | Insomnia | Infusion drunk |
| **Pedaliaceae** |  |  |  |  |  |  |
| *Sesamum indicum* L., (BTH099), 1 | Ekannu (Lun) | 1 | S | Fruit | Hypogalactorrhea | Fried seed eaten |
| **Phytolaccaceae** |  |  |  |  |  |  |
| *Phytolacca dodecandra* L'Herit., (BTH084), 15 | Ehilopolopo (Lun) | 3 | S | Leaf | Itchy skin | Infusion bathed |
|  |  | 12 |  | Leaf | Cutaneous mycoses | Poultice applied topically |
| **Plumbaginaceae** |  |  |  |  |  |  |
| *Plumbago zeylanica* L., (BTH076), 5 | Kacekere (Lun) | 5 | H | Shoot | Ulcers | Decoction of powder drunk |
| **Poaceae** |  |  |  |  |  |  |
| *Cymbopogon citratus* (DC) Stapf, (BTH095), 4 | Kyai subi (Lun) | 4 | H | Leaf | Flu | Steam bath, decoction drunk |
| *Cynodon dactylon* (L.) Pers. (BTH088), 9 | Olufaafa (Lun) | 2 |  | Leaf | Diarrhea | Infusion drunk |
|  |  | 7 |  | Shoot | Fever | Steam bath |

**Table. S1 continued**

| Family, scientific name,  (Voucher number), TFM | Local name | FM | HB | Part used | Ailment | Mode of administration |
| --- | --- | --- | --- | --- | --- | --- |
| *Melinis repens*(Willd.) Zizka, BTH094), 2 | Nawunyasi (Lun) | 2 |  | Leaf | Cancer | Decoction drunk |
| **Polygonaceae** |  |  |  |  |  |  |
| *Oxygonum sinuatum* (Meisn.) Dammer, (BTH077), 5 | Nkenge (Lun) | 5 | H | Leaf | Ulcers | Infusion drunk |
|  |  | 2 |  | Leaf | Malaria | Infusion bathed |
| *Rumex usambarensis* (Dammer), (BTH079), 28 | Nankombi (Lun) | 5 | H | Tuber | Cutaneous mycoses | Poultice applied topically |
|  |  | 13 |  | Tuber | Pressure | Decoction drunk |
|  |  | 4 |  | Leaf | Diabetes | Infusion drunk |
|  |  | 2 |  | Root | Colic in babies | Decoction drunk |
|  |  | 1 |  | Stem bark | Backache | Decoction drunk |
|  |  | 3 |  | Stem bark | Stomachache | Decoction drunk |
| **Portulacaceae** |  |  |  |  |  |  |
| *Talinum paniculatum* (Jacq.) Gaertn., (BTH097), 32 | Eposa (Lun) | 7 | H | Shoot | Dystocia cervix | Sitting in the infusion |
|  |  | 21 |  | Shoot | Witchcraft | Poultice applied on the face |
|  |  | 4 |  | Shoot | Sceptic ears | Drop extract in ears |
| **Rhamnaceae** |  |  |  |  |  |  |
| *Gouania longispicata* Engl. (Ait.) Steud., (BTH078), 5 | Namayendeyende (Lun) | 3 | S | Leaf | Cutaneous mycoses | Concoction drunk |
|  |  | 2 |  | Leaf | STIs | Concoction drunk |
| *Ziziphus abyssinica* A. Rich., (BTH087), 2 | Namukodolya (Lun) | 2 | S | Root | Hydrocele | Infusion drunk |
| **Rutaceae** |  |  |  |  |  |  |
| *Citrus limon (*L.) Burm.f. (BTH085), 23 | Eniimu (Lug) | 15 | T | Leaf | Cough | Decoction drunk |
|  |  | 8 |  | Fruit | Cough | Juice extract mixed with hot water and drunk |
| *Citrus sinensis* (L.) Osb., (BTH093), 3 | Mucungwa (Lug) | 3 | T | Leaf | Malaria | Steam bath |
| **Rubiaceae** |  |  |  |  |  |  |
| *Coffea canephora* Pierre ex A. Froehner, (BTH102), 25 | Emwanyi (Lug) | 6 | S | Leaf | Chest pain | Concoction drunk |
|  |  | 19 |  | Fruit | Erectile dysfunction | Decoction drunk |
| *Rubia cordifolia* L., (BTH0125), 1 | Salabagesi (Lun) | 1 | H | Leaf | Cough | Salt added to ash and licked |
| *Sarcocephalus latifolius* (Smith) Bruce, (BTH098), 9 | Omutamatama (Lun) | 9 | T | Root | Osteodynia | Decoction drunk |
| *Spermacocce princei* (K.Schum.) Verdc., (BTH0116), 1 | Nafi (Lun) | 1 | H | Leaf | Bone fracture | Poultice applied topically |
| **Rutaceae** |  |  |  |  |  |  |
| *Teclea nobilis*Hook.f. (BTH0124), 2 | Omugangwe (Lun) | 2 | S | Root | Osteodynia | Decoction drunk |
| *Zanthoxylum chalybeum* Engl., (BTH0104), 18 |  | 7 | S | Root | Candidiasis | Concoction dunk. |
|  |  | 11 |  | Root | Measles | Boiled in milk and drunk |
| *Zanthoxylum* *leprieurii* Guill. & Perr., (BTH0115), 16 | Mutaninwa (Lun) | 12 | T | Root  Leaf | Gastro intestinal complications | Decoction drunk |

**Table. S1 continued**

| Family, scientific name,  (Voucher number), TFM | Local name | FM | HB | Part used | Ailment | Mode of administration |
| --- | --- | --- | --- | --- | --- | --- |
|  |  | 4 |  | Root | Fever | Powder mixed with food and eaten |
| **Simaroubaceae** |  |  |  |  |  |  |
| *Harrisonia abyssinca* Oliv. (BTH111), 21 | Njeta (Lun) | 20 | T | Leaf | Measles | Powder mixed with ghee and applied topically |
|  |  | 1 |  | Root | Gonorrhea | Decoction drunk |
| **Solanaceae** |  |  |  |  |  |  |
| *Capsicum frutescens* L*. (*BTH0117), 21 | Kamulali (Lug) | 3 | H | Root | Aphrodisia | Baked in hot ash, chewed and extract swallowed |
|  |  | 9 |  | Root | Dystocia labor | Chewed and extract swallowed |
|  |  | 6 |  | Root | Erectile dysfunction | Infusion of powder drunk |
|  |  | 3 |  | Fruit | Visual impairment | Mixed with food and eaten |
| *Nicotiana tabacum* L. (BTH115), 14 | Nahataba (Lun) | 3 | H | Leaf | Candidiasis | Decoction drunk |
|  |  | 11 |  | Leaf | Snake bite | Extract applied topically on the wound |
| *Physalis peruviana* L., (BTH118), 4 | Ntutunwe (Lun) | 4 | H | Leaf | Vomiting | Infusion drunk |
| *Schwenkia americana* L., (BTH123), 2 |  | 2 |  | Leaf | Spirit possession | Smoke burnt leaves inhaled |
| *Solanum aethiopicum* L., (BTH105), 3 | Nakati (Lug) | 3 | S | Fruit | Ulcers | Decoction drunk |
| *Solanum anguivi* Lam***.,* (**BTH122), 24 | Kantuunkuma (Lug) | 24 | S | Fruit | Hypertension | Steamed and eaten |
| *Solanum gilo* Raddi, (BTH106), 1 | Enjagi (Lun) | 1 | S | Fruit | Hypogalactorrhea | Boiled and eaten |
| *Solanum lycopersicum* L., (BTH121), 10 | Enyanya (Lug) | 10 | H | Leaf | Vomiting | Infusion drunk |
| *Solanum tuberosum* L., (BTH107), 10 | Obumonde (Lug) | 13 | H | Tuber | Ulcers | Infusion drunk |
| *Solanum campylacanthum*Hochst. ex A. Rich., (BTH0113), 25 | Ntengontengo (Lug) | 24 | S | Root | Erectile dysfunction | Chewed and extract swallowed/decoction drunk |
|  |  | 1 |  | Root | Hydrocele | Infusion drunk |
| **Theaceae** |  |  |  |  |  |  |
| *Camellia sinensis* L., (BTH130), 4 | Amajaani (Lug) | 4 | S | Leaf | Gastro intestinal complications | Infusion of the powder drunk |
| **Verbenaceae** |  |  |  |  |  |  |
| *Clerodendrum myricoides* (Hochst.) Vatke, (BTH0126), 15 | Akhanghololo (Lun) | 5 | S | Leaf | Cough | Ash licked or chewed and extract swallowed |
|  |  | 9 |  | Leaf | Snake bite | Ash licked or chewed and extract swallowed |
|  |  | 1 |  | Root | Indigestion | Infusion drunk |
| *Lantana camara L.*, (BTH0112), 19 | Kapanga (Lun) | 13 | S | Leaf | Itchy eyes | Eyes washed with infusion |
|  |  | 6 |  | Leaf | Malaria | Infusion bathed |
| *Vitex ferruginea* Schumach. & Thonn., (BTH0133), 6 | Muheremba (Lun) | 4 | S | Leaf | Sore throat | Infusion drunk |
|  |  | 2 |  | Leaf | Cough | Chewed while holding hands at the back and swallow the extract |
| **Vitaceae** |  |  |  |  |  |  |
| *Cyphostemma adenocaule* (A. Rich.) Wild & R.B. Drumm., (BTH0114),12 | Nawagajo (Lun) | 5 | H | Leaf | Breech position in pregnancy | Infusion drunk |

**Table. S1 continued**

| Family, scientific name,  (Voucher number), TFM | Local name | FM | HB | Part used | Ailment | Mode of administration |
| --- | --- | --- | --- | --- | --- | --- |
|  |  | 7 |  | Leaf | Retained placenta in humans | Infusion drunk |
| **Zingiberaceae** |  |  |  |  |  |  |
| *Cucurma longa* L., (BTH0127), 26 | Ekinzaali (Lug) | 12 | H | RH | Diabetes | Decoction of powder drunk |
|  |  | 4 |  | RH | Liver | Powder mixed with food and eaten |
|  |  | 3 |  | RH | Breast | Powder with food and eaten |
|  |  | 3 |  | RH | Uterine fibroids | Powder with food and eaten |
|  |  | 4 |  | RH | Pressure | Decoction drunk |
| *Zingiber officinale* Rosc, (BTH120), 16 | Entangauzi (Lug) | 13 | H | RH | Toothache | Concoction applied on tooth |
|  |  | 3 |  |  | Oesophageal cancer | Concoction drunk |
| **Zygophyllaceae** |  |  |  |  |  |  |
| *Tribulus terrestris* L., (BTH119), 3 | Nkenge omunene (Lun) | 3 | H | Leaf | Septic ears | Extract dropped in ears |
|  |  |  |  |  |  |  |
| **Unidentified** |  |  |  |  |  |  |
| (BTH131), 2 | Mutasuwa (Lun) | 2 |  | Root | Backache | Infusion drunk. |
| (BTH129), 1 | Enjuge (Lun) | 1 |  | RH | Gonorrhea | Decoction drunk |
|  |  | 1 |  | Root |  | Infusion drunk |
| (BTH128), 1 | Omughongha (Lun) | 1 |  | Tree | Swollen legs due to witchcraft | Poultice applied topically |
| (BTH132), 1 | Murembe (Lun) | 1 |  | Leaf | Cough | Decoction mixed with honey and drunk |

**Key:** Lun; Lunyole, Lug; Luganda, TFM; Total frequency of mention FM; Frequency of mention, HB; Habit, H; Herb, S; Shrub, T; Tree, RH; Rhizome; WP; Whole plant
